# Self-tracking in endometriosis: Evolving expectations around a gynecological app developed by a Finnish patient organization

**DOI:** 10.1177/03063127251344961

**Published:** 2025-06-14

**Authors:** Venla Oikkonen, Maria Temmes

**Affiliations:** Tampere University, Tampere, Finland

**Keywords:** chronic illness, digital health, endometriosis, expectations, patient organization, self-tracking

## Abstract

The article examines the possibilities and limits of patient advocacy-led health technology design through a case study: a non-commercial mobile self-tracking app developed by a Finnish patient organization to advance medical care and research in endometriosis, an underfunded and understudied gynecological condition. Drawing on interviews with patient organization representatives, specialized clinicians and people with endometriosis, as well as written endometriosis stories, this article traces the evolving expectations around the app to understand the landscape of hopes and concerns in which patient advocacy-led design is conceived and received. This article identifies tensions in visions about how the app could be used as well as locates shifts in expectations as the app moved from an idea to everyday use. The article also shows how structural aspects of established technological systems, such as digital health infrastructures or data ownership relations, shape expectations about future uses of patient advocacy-led technology. This case study contributes to science and technology studies scholarship on self-tracking and health technology development by providing a nuanced understanding of how the dynamics of expectation in patient advocacy-led design operate in a complex and underdiagnosed gendered chronic illness.

Science and technology studies (STS) scholars have noted that digital technologies, often developed and maintained by private companies, have an increasingly central role in public healthcare institutions (Faulkner-Gurstein & Wyatt, 2023; Fox, 2023). This stresses the importance of understanding what kinds of technologies are integrated into the healthcare infrastructure and how it happens. At the same time, social scientists have noted that patient participation in healthcare infrastructures and health technology development has emerged as an object of expectations in healthcare policies and strategies (Gentilini & Miraldo, 2023; M. Jones & Pietilä, 2018; Marent et al., 2023). However, concerns have been raised that technology companies interested in ‘scaling’ their technologies engage in ‘participation washing’ by ignoring the context-dependent nature of participation and the complexities and ambiguities of user experience among diverse, situated populations (Sloane et al., 2022).

This article explores what happens to expectations about health technology development when a patient advocacy community, rather than the usual commercial or institutional actors, envisions, develops, and launches a novel health app. We focus on a non-commercial self-tracking app developed for endometriosis by a gynecological patient organization in Finland. Endometriosis is a chronic gynecological condition in which tissue similar to the lining of the uterus grows outside the uterus, typically in the pelvic or abdominal cavity. Endometriosis reacts to changes in estrogen levels, which cause inflammation at the site of endometriosis lesions, often resulting in severe pain (Horne & Missmer, 2022).

The organization initially envisioned the app as a means of addressing gendered biases in healthcare by reducing diagnostic delays in endometriosis and allowing users to follow the progression of their illness. After its launch, the app underwent further development in which its potential userbase was expanded to include other gynecological conditions, with plans to integrate it into the institutional structures of endometriosis care and research. The case provides a unique viewpoint into the evolving, situated dynamics of expectations in patient advocacy-led health technology in a complex and under-researched chronic illness.

Our exploration of the visions, hopes, and concerns around the app is inspired by the rich science and technology studies and sociological literature on expectations. This literature suggests that expectations are often mobilized in ways that enact new sets of connections between different actors, interest groups, and sites of technology use across biomedicine, technology, and society (Borup et al., 2006). While expectations are social constructs, they have material effects. Promissory discourses around biotechnological innovation mobilize actors and attract economic interest towards certain lines of development (Petersen & Krisjansen, 2015). They are also performative in that they legitimize research initiatives, or value some research practices over others (Borup et al., 2006; Flear, 2021). At the same time, expectations evolve over time and may move in different directions at different sites, resulting in tensions (Borup et al., 2006; Brown & Michael, 2003). Hence, expectations require maintenance and possible reframing from their advocates. This means adjusting the broad societal framework within which new biotechnological practices are promoted (Tarkkala et al., 2019) as well as negotiating the inevitable gap between expectation and reality in the day-to-day practices of clinical work with patients (Gardner et al., 2015).

Previous research has shown that in health policy, self-tracking technologies embody expectations of increasing the economic sustainability of health services, especially in managing chronic conditions (Morgan, 2016). Yet, in practice, incorporating patient produced data has proven tricky in clinical care and research, and expectations are far from uniform or fixed (Haase et al., 2023; Vegter et al., 2021). This points to the plurality of expectations: Even when an expectation is shared by many on a policy level, it is often reframed or contested at the sites where technologies are mobilized in practice (Gardner et al., 2015). Furthermore, expectations operate with multiple registers, ranging from the carefully phrased statements of policy documents to fleeting feelings of hope and concern in everyday uses of technologies. In this article, we focus on how expectations operate on the more mundane and practical end of this spectrum, while including both communal and personal visions, concerns, and hopes about patient advocacy-led technology development.

In this article we unpack the range of premises underlying the expectations of the patient organization, clinicians, and endometriosis patients. These expectations were not the same: while the patient organization put considerable effort into developing the app with a growing set of expectations attached to the technology, patients’ and clinicians’ expectations focused on its practical usefulness in living with or treating endometriosis. Our analysis charts the intersections of these expectations. To understand the evolving dynamics of expectations, we ask: (1) To what extent was the patient advocates’ hope—that the app would help capture the specificities of endometriosis— realized, and what complicated its realization in practice? (2) What uses did different actors, across different contexts of use, envision for the app and its data? (3) How have structural features of technological systems shaped expectations about the future of the app? In exploring these questions, we pay special attention to how expectations differ across contexts of design and use, as well as how they change within each context. This allows us to trace both emerging patterns and tensions across sites.

## Self-tracking, menstruation, and chronic illness

Endometriosis is a chronic gynecological illness that is estimated to affect around 10% of women globally (WHO, 2023), and also is a health concern among transgender people (Griffith, 2019; Jeffrey et al., 2024). While its first symptom is usually prolonged and severe pain during periods, the symptoms often expand over years to include, for example, gastrointestinal pain and bleeding or pain during exercise or sex—symptoms that occur also outside periods (Griffith, 2019; Hudson, 2022; Seear, 2014). In biomedicine, endometriosis is often described as an estrogen-dependent disease, yet the exact mechanisms behind endometriosis-related pain are still poorly understood (Grundström et al., 2019). Furthermore, endometriosis is increasingly considered as a chronic systemic disease, with endometriosis lesions found in various parts of the body, including the lungs and the brain (Taylor et al., 2021). Previous feminist research has also emphasized that the link between menstruation and endometriosis is not straightforward, and endometriosis research requires a more versatile basis to think about the multiplicity of symptoms (C. E. Jones, 2015).

The complexity and rarity of many endometriosis symptoms provides a rationale for using self-tracking tools: Knowing one’s body and being able to distinguish between different types of pain symptoms may help in assessing and managing symptoms (Helosvuori & Oikkonen, 2024). While the typical worsening of endometriosis symptoms during estrogen fluctuations aligns endometriosis with period and ovulation tracking technologies, commercial period tracking apps are primarily marketed as means of monitoring spontaneously occurring cycles that are not shaped by hormonal contraceptives use (Della Bianca, 2021). As endometriosis is commonly treated with hormonal products to control hormonal levels and halt menstruation, period tracking apps are limited in their capacity to aid treatment of endometriosis. The occurrence of endometriosis symptoms outside menstruation further curtails the usefulness of period tracking apps.

The patient organization positioned their app in relation to the expanding field of commercial period and ovulation tracking apps. Such technologies promise to predict menstrual cycles based on information about previous cycles and other health information, indicating either the safest days for sex to prevent pregnancy or, alternatively, an ideal time for conception. Researchers have shown that period and ovulation trackers are used creatively for a multitude of goals in situated practices, some of which challenge assumptions that structured the initial design of these technologies (Algera, 2023; Della Bianca, 2021; Hamper, 2020). This is in line with findings across different self-tracking technologies and practices, which suggest that users choose what, when, and how they track, and may engage with only some of the data produced through the app (Pantzar & Ruckenstein, 2017; Roberts et al., 2019; Weiner et al., 2020). In the case of chronic gynecological conditions, those using commercial period tracking apps may interpret the data in relation to their illness. For example, what constitutes an expected cycle for someone with endometriosis or polycystic ovarian syndrome (PCOS) might be a concerning sign for someone tracking their period for contraception. Our analysis shows that the app developed by the patient organization departs from most commercial period tracking apps in that it builds on the notion that bodies are shaped by biomedical practices, such as medications or surgical procedures, and that bodies respond uniquely to both illness and its treatment.

The uses of period tracking apps are expanding beyond monitoring menstrual cycles, for example, to embrace a broader idea of ‘hormonal health’ (Ford et al., 2021) or to prepare for potential future problems with fertility (Roberts & Waldby, 2021). The European Society of Human Reproduction and Embryology guidelines for endometriosis treatment suggest that tracking symptoms can be useful for endometriosis patients to make their pain visible in the clinic (Becker et al., 2022). While this expansion may reflect users’ personal hopes and concerns, self-tracking technologies also place the responsibility for seeking health onto individuals, who are expected to respond to the data produced through health technologies and engage in further modes of self-tracking (Lupton, 2016; Roberts & Waldby, 2021; Till, 2019; Weinberg, 2021). Researchers have also highlighted concerns about data privacy in this commercially-led field, for example, whether the personal data produced through a self-tracking app could be mobilized against the person using the app (Lupton, 2016; Neff & Nafus, 2016). Our analysis shows how the patient organization framed their app as residing outside the logic of commercialization in response to these concerns.

We approach the patient organization’s app also in relation to self-tracking in chronic illness. In healthcare policies, self-tracking is often envisioned as a cost-effective means of identifying the appearance or progression of health conditions (Haase et al., 2023). The premise is that self-tracking technologies enable users to trace changes in health outside the doctor’s office and act proactively before a health concern materializes as a life-threatening condition (Haase et al., 2023; Neff & Nafus, 2016). However, incorporating digital health technologies into the siloed structures of healthcare systems is not easy, for example, disconnections between sites of care interrupt the flow of data between people or units (Lupton, 2017). Healthcare providers also see potential problems in how the technologies are used at home or how they may shape the patient-doctor relationship (Fiske et al., 2020; Ruckenstein, 2015).

Using self-tracking technologies in chronic illness raises specific questions. While some chronic conditions, such as type 1 diabetes, threaten life without constant tracking (Mialet, 2022), the benefits are more ambiguous in other conditions. Self-tracking may help patients recognize what triggers their symptoms, but the continuous charting of symptoms may also make chronic illness feel constantly present (Bagge-Petersen, 2023; Lehoux, 2008; Marent et al., 2018). This ambivalence, between learning to understand one’s illness and knowing too much, is also present in the user experiences we explore in our analysis of the patient organization’s app. Crucially, self-tracking as a practice can appear never-ending (Bergroth, 2019; Till, 2019), and documenting intimate aspects of life (sex, moods, etc.) may feel invasive (Hamper, 2020). This dynamic is enhanced in chronic illness, where managing illness may already feel like work. Furthermore, if self-tracking is directed at a biological process that is beyond the control of the user, such as menopause, self-tracking may appear pointless (de Boer et al., 2023).

In recent years, there has been growing interest in developing digital health technologies in collaboration with users, patient advocates, health care professionals, and developers (e.g., Lupton, 2017; Marent et al., 2018). Participatory design, or designing with stakeholders, has been seen as a way of addressing the long-standing concern that technology design tends to ignore the needs of the users: patients and healthcare providers. Researchers have documented experiences from participatory design workshops that aim to envision future digital health technologies (Lupton, 2017) or their potential applications in chronic conditions (Gardsten et al., 2017; Marent et al., 2018). Such workshops often encourage participants to imagine how technologies might be used (e.g., Marent et al., 2018). The app we study differs from participatory design projects. While the patient organization sought to engage people with endometriosis as well as clinicians at crucial points of the development, the organization was responsible for envisioning the app and making decisions about its realization. Our study also differs from future-oriented accounts of participatory design in that we show how visions evolve when an app becomes a material reality.

## Data and methods

This article is part of a social science research project on gendered chronic illness in Finland. The data was collected in an endometriosis subproject where one focus was the uses of the app in tracking endometriosis symptoms. Although the app is currently promoted as a means of managing several gynecological conditions, it was initially developed to track endometriosis symptoms, and people with endometriosis remain its largest user group.

Our analysis draws on an interview dataset that includes four interviews with members of the patient organization, 13 interviews with healthcare professionals, and 16 interviews with people with endometriosis, all conducted in Finland in 2021–2023. We obtained written informed consent from all the interviewees. The collection and analysis of the data follows the ethical guidelines of the Finnish National Board on Research Integrity.

The four representatives of the patient organization have each held a formally recognized position within the organization involving leadership, coordination, or planning responsibilities. They have followed the development and use of the app at different stages. To maintain their anonymity, details about their precise role within the organization have been removed. We located the healthcare professionals by identifying key actors in endometriosis care, who, in turn, referred us to other healthcare professionals specialized in endometriosis. Some interviews with healthcare professionals also took place in the context of a two-week visit to a public endometriosis clinic. The interviewed healthcare professionals include gynecologists, endometriosis nurses, surgeons, pain specialists, and sexual counsellors at public hospitals. The use of the app was discussed by seven of these interlocutors. To guarantee anonymity, we refer to our interlocutors as healthcare professionals, or, when important for analysis, use general professional categories (e.g., endometriosis nurse, sexual counsellor).

For the interviews with people with endometriosis, we found our interlocutors through a call for research participants posted on our project’s website and Twitter account, and circulated through the patient organization’s networks. The use of digital channels for recruitment was appropriate, as our interlocutors were members of digitally competent patient groups, that is, the same groups that are also seen as potential users of the app. Using digital platforms in data collection enabled us to reach people with endometriosis across the country, including areas with no specialized endometriosis clinics. While the call was open to any gender, our interlocutors are mostly cisgender women. They all have an endometriosis diagnosis based on imaging tests, surgery findings, or symptoms. Among the people with endometriosis we interviewed, many had illness experiences that preceded the development of the app and, at the time, some did not have symptoms and thus had no need for symptom tracking. Yet all except two knew about the app, and eight had used it. Furthermore, several of our interlocutors used other digital apps for activity, wellness, and dietary symptoms and saw the patient organization’s app as a part of the larger field of self-tracking apps they engaged with.

Semi-structured interviews were conducted by Oikkonen with people with endometriosis, and by Temmes with patient advocates and healthcare professionals. The interviews with the patient organization included extensive discussion on the development of the app and the ways in which the app fits within the broader strategies of the organization. The interviews with the healthcare professionals addressed the app in the context of the use of digital technologies in endometriosis care. The interviews with people with endometriosis inquired about the role of self-tracking tools in general, and the patient organization’s app in particular, in the daily management of endometriosis.

In addition to the interviews, we draw on 43 anonymous written stories of living with endometriosis collected through an online form on the project’s website. Our call for stories invited people with endometriosis to write about any aspect of their illness experiences and mentioned the app as one possible topic. The length of the stories varies between a short paragraph and several pages. Nine of the stories discuss the app. When discussing the experiences of people with endometriosis, we indicate after each quote whether it is from an interview or written story.

The analysis was conducted jointly without specialized software. We identified mentions of the app as well as of digital health technologies generally. This was followed by two lines of comparison. First, we located differences in expectations over time within specific settings, such as the patient organization and healthcare facilities specializing in endometriosis. Second, we read the mentions of the app across patient advocacy, clinical practices, and everyday illness experiences to identify both differences and similarities in expectations about the app. Furthermore, we paid attention to the material and institutional conditions that enabled the development of the app, as well as the structural challenges that slowed the full realization of some of its most ambitious goals. This allowed us to explore the ways in which expectations are entangled not only in visions of endometriosis care but also in the structural preconditions that constitute technologies as feasible or unfeasible.

## Introducing the case study

The creation of the app started in 2016 as part of a project receiving money from the state-run funding system for NGOs, currently known as the Funding Centre for Social Welfare and Health Organizations. The project sought to raise awareness about endometriosis among young people to reduce the diagnostic delay for endometriosis, which is estimated to be six to nine years in Finland. Feminist research on endometriosis notes that the diagnostic delay is linked to the gendered biases in healthcare as severe pain during menstruation is often dismissed as normal (Hudson, 2022; Seear, 2009). Addressing the existing gendered biases in endometriosis treatment and research has been one of the main aims of the patient organization. The app was designed to help users recognize the extent of their symptoms and gather evidence that could be shown to clinicians. While the development of the app was not the focus in the project, the funding enabled the patient organization to hire a person to lead the new project and contract a company to help in creating and maintaining the technological functions of the app. Expectations were tentative at this point: The app was seen as one means (among many) of reaching young people with diagnosed endometriosis or symptoms consistent with endometriosis.

The motivation for the app arose from the observation that commercial period tracking apps were not very useful for those who were on hormonal medication or had complicated symptoms not aligned with menstrual bleeding (such as gastrointestinal problems). The need for an app was recognized across endometriosis patient advocacy communities. One of the patient organization representatives recounted that the topic was raised in the Nordic Endometriosis Association in 2013. The possibility of creating an app that could be used in the Nordic countries was discussed, but the plans were paused due to a lack of resources. One representative noted in an interview that this led the Finnish patient organization to decide that instead of waiting, they would ‘push this through in Finland’.

To fulfill expectations about the app’s usefulness for people with endometriosis, the patient organization drew on its established connections with trusted clinicians specializing in endometriosis. The patient organization representatives mentioned in interviews that the collaboration with clinicians set the app apart from commercial self-tracking apps. One representative noted that while commercial apps seek to make profit through ads or paid extensions, the patient organization was ‘not selling the app or making profit with it’ and was ‘considered to be reliable because we collaborate with healthcare professionals and specialist doctors.’ This role of the organization was considered to offer the app legitimacy amongst people with endometriosis.

Data ownership and privacy were seen as another central feature distinguishing the patient organization’s app from commercial products. One representative noted that in commercial apps, ‘you have to give up a lot of data and rights’ whereas the patient organization owns the data in their app and would not ‘collect any unnecessary information, with which we’d do nothing’. The personal data collected in the app, stated in the app’s privacy statement, consists of email address, password, and voluntary information such as full name, weight, height, and any health information the user saves to the app. While the privacy statement notes that the company who provides technical services to the organization might need to handle some personal data, it emphasizes that no data is handed to any other third parties without a separate consent from the user. One representative also noted during the interview that the organization does not collect any statistical information, although a separate consent form could enable such gathering for research or treatment purposes. From the early stages of the app development, data protection and users’ right to control personal data were envisioned as a key strength of the app. This included the right to know what data is being stored and to ask for the removal of data.

## Capturing endometriosis symptoms

From its beginnings, the patient organization’s app project was designed with an expectation that the app should track the complexities of endometriosis. The patient organization saw that the focus on endometriosis symptoms would distinguish the app from commercial period tracking apps. One of the patient organization representatives noted, in an interview: ‘When period tracking apps started to appear we thought immediately that these are not fit for our purposes, not if you have a medication that causes you not to menstruate.’ She outlined the organization’s ‘completely different starting point that acknowledges the multifaceted nature of endometriosis symptoms’:

You can follow your symptoms regardless of whether you are bleeding at all or irregularly. And even if you are bleeding, symptoms can appear also outside of bleeding because people with endometriosis might have pain related to ovulation that is very different compared to other people. So [the new app] takes into consideration that not everyone bleeds. Or if we think of people whose womb has been removed, they still have hormonal cycles if they still have ovaries.

In contrast to commercial period tracking apps, the patient organization based its app on the understanding that the absence of menstruation does not eliminate the need to track symptoms, thus stressing the importance of disentangling the tracking of endometriosis symptoms from menstrual bleeding. The vision behind the app was to capture the peculiarities of bodies shaped by endometriosis and potentially undergoing hormonal treatment for endometriosis.

However, capturing an illness with complex and diverse symptoms is a complicated task. Apps focus on a selected set of variables—in this case, symptoms—that are considered to be the most important for the tracked phenomenon, such as a health condition. The selection of traceable symptoms in turn provides the data that can be used to follow the progression of illness or the effectiveness of medication over time. The patient organization representatives we interviewed highlighted the significance of carefully selecting symptoms for the app. In addition to information that might be useful for most of the users such as pain symptoms, menstrual and other types of bleeding (such as breakthrough bleeding during the hormonal medication), pain medication, and work absence, the organization needed to consider whether to include less prevalent symptoms. This task became particularly challenging in 2019, when the organization expanded from being an endometriosis organization to become a gynecological patient organization, representing two other gynecological conditions. With the expansion, the traceable symptoms, which had initially focused on endometriosis, were re-evaluated with the new patient groups in mind. It was considered important to focus on symptoms that were understood to be the most informative ones about the progression of each illness.

Multiple, not-fully-compatible expectations existed around the question of what exactly the app should capture. Already with endometriosis there is a lot of variation in symptoms between people, leading to an inevitable gap between tracked symptoms and the embodied complexities of a particular case of endometriosis. One of the patient organization representatives noted that many people ‘have their own specific symptom’ not shared by most users of the app, which makes design decisions difficult:

I understand that from the user’s viewpoint it would be wonderful to trace the symptom, but that always brings the challenge of balancing between having an app that is sufficient, with enough information and things that you can follow, and not making it too complicated.

Even a specialized app for a chronic condition needs to seek a balance between capturing the embodied experiences of an illness and the ease of daily use. To maintain and finetune that balance, the patient organization remained open to the possibility of modifying the traceable symptoms based on user feedback. The organization collected feedback with separate calls that they advertised on their own online channels. This was done, for example, when symptoms for other gynecological conditions were added to the app and when the app underwent an update. One representative noted, in an interview, ‘we want to follow how people have started to use the app, what they think and like about it’.

The case of ovarian pain demonstrates this tricky dynamic of inclusion and exclusion. Despite users expressing hopes that ovarian pain would be included in the app, it has not been added there. A patient organization representative explained the organization’s reasoning:

We have pain in the stomach and pelvic area there, but some would like it to be more specifically about an ovary, that it’s hurting. We have discussed and considered it but think that it might not be that central where the pain is—it may be central for the individual but not necessarily from the viewpoint of healthcare.

Here the concern about the ease of use is merged with the concern about the medical usefulness of including a specific symptom. This suggests that expectations about the app’s ability to capture endometriosis symptoms were complicated by multiple coexisting rationales of use, the clinical usefulness of the data being one central consideration. Furthermore, the organization still shares a paper-based symptom diary on their website. The diary offers the possibility of tracing symptoms—such as ovarian pain—that reflect rare illness experiences. In such situations, the open-endedness of paper-based symptoms-diary may be preferred by users over the quantitative logic of the digital app with its inbuilt exclusions.

In addition to capturing symptoms at present moment, self-tracking of a health condition is expected to capture gradual changes in illness. Endometriosis is a chronic—and, in many cases, progressive—illness, and chronic illnesses have their own distinct temporalities that span over years. Many of our interlocutors with endometriosis appeared to have started using the app with relatively few expectations, yet they expressed hopes that the app would engender patterns of endometriosis symptoms over time. Such patterns are seen as having a potentially important function as a source of medically useful information. For example, one of our interlocutors described realizing through the pattern produced by the app that her symptoms have changed:

I realized now when I filled out information in the app that I have not really had defecation pains at all, and they used to be really, really severe when I was younger, for example, when I was 15. Now I realized that I haven’t had them at all. So something has happened. Somehow, when they are marked there, and you see directly in the calendar view when you have not had pain and when you have. (Interview, early 20s)

The pattern produced by the app is experienced here as potentially liberating as it points to an absence of a previously debilitating symptom. In this instance, the app appears as a technology that turns an unnoticed absence into a quantified reality, making significant aspects of endometriosis visible.

Importantly, this sense of capturing endometriosis is produced through a discrepancy between the embodied personal history of chronic illness and the data produced through the app. The data that the app used is based on the users’ own experiences of daily variations in symptoms. At the same time, the app produced a different understanding of endometriosis than the one conceptualized by the user before using the app. The distance that appears between embodied illness experience and pattern-oriented digital data engenders moments of surprise and potential confusion. For example, the app might generate a distinct pattern of symptoms over a period of time that does not correspond to the user’s experience of a chaotically undulating illness. This points to an epistemic tension between the pattern-oriented logic of self-tracking and the unpredictable shifts in symptoms experienced by many people with endometriosis. This tension characterizes the relationship between quantification and embodied experience in general: The self-tracking literature has explored how people navigate across different types of sensory and digital modes of knowledge as they engage in self-tracking (Algera, 2023; Bergroth, 2019; Della Bianca, 2021; Lupton, 2016). Our data suggests that such tension may be heightened in chronic, progressive illnesses, as the temporality of a potentially progressive illnesses adds a sense of urgency to self-tracking.

Expectations about the app’s ability to capture the temporality of endometriosis as a chronic, progressive illness also centered on how the app differs from the built-in cyclicality of a commercial period-tracking app. By tracking gradual changes in symptoms, medications, and bleeding—including menstrual, pharmaceutically induced, as well as unexplained bleeding—over time, the app may point to slowly emerging changes in the chronicity of endometriosis that override a clear sense of hormonal cyclicality. For several interlocutors with endometriosis, perceiving patterns of symptoms over time was a welcomed outcome of using the app. Yet there are also many who had engaged selectively with the data produced through the app. One interlocutor with a history of endometriosis, migraine, and different experiments of tracking pain, explained how monitoring one’s symptoms does not necessarily result in a heightened sense of an illness trajectory:

When I’m not in pain, I may forget all those times [when I was in pain] because I don’t have pain. I’m not interested in monitoring pain or in the pain itself. I’m not attached to it. It’s rather the opposite with me. When I experience a period of time without pain, I forget that my body is a pain patient’s body. (Interview, late 40s)

This user chooses to ignore the pattern established by the app and the sense of chronicity it implies if doing so helps distance the illness trajectory even momentarily. Such selective use deviates from the expectation that capturing endometriosis symptoms through self-tracking is beneficial.

One of our interlocutors with endometriosis also pointed out that if the symptoms of endometriosis are continuous, the app might not be able to detect any pattern, as the app ‘becomes so full of notes that it’s not useful’ (Written story, mid-40s). In other words, in some cases of endometriosis, there may be no discernable pattern in symptom manifestation over time, even if the user wishes to track it. Among several interlocutors, such lack of pattern confirmed the users’ sense that their endometriosis is ever-present. This resonates with Hamper’s (2020) observation in the context of fertility tracking that it can be daunting if an app is not able to identify any fertile windows, thereby highlighting implicit pathologies. The underlying temporality of a progressive illness like endometriosis adds to how the app’s inability to distinguish a pattern may be felt. The apparent ever-presence of symptoms and lack of a distinguishable pattern may raise concerns about the future direction of the illness.

## Different visions of how to use the app

The app was also surrounded by multiple expectations about the ways in which the data produced through it could be used. The app’s design project was based on the premise that tracking symptoms can be important in seeking and receiving adequate endometriosis care. According to a patient organization representative, one motivation for developing a self-tracking app arose from ‘cursing the old printable symptoms stuff’. While the printable symptom diaries offer ways of marking down different types of pain and medications, and as noted above allow tracking rare symptoms, the paper-based mode of tracking was seen as ‘horribly laborious’, as one patient organization representative put it. She explained:

It makes you feel also mentally bad if you must focus on and write down for a doctor how much you’re hurting today and really wallow on the fact that you’re having a shitty day. It’s awful and takes a lot of mental energy. If you can get from writing these down in five to 15 minutes every day to being two minutes on your phone, click click click, it helps as it’s done faster. You don’t have to carry anything with you. You can add things during your school or workday. If your period starts in the middle of the school day, you can just click.

Time is understood here to be more than just a matter of effective time management when tracing symptoms that might be useful for managing endometriosis. It is important because it means spending less time thinking about the symptoms. While previous research on self-tracking in chronic illness documents concerns that self-tracking could increase the felt presence of chronic illness (Bagge-Petersen, 2023; Marent et al., 2018), in the excerpt above the gynecological app is envisioned as reducing anxieties. The quote suggests that the app was seen as producing a record of symptoms while allowing the users to distance themselves from the presence of the chronic illness.

Among our interlocutors with endometriosis, many recognized these expected benefits of the app and, as seen in the previous section, highlighted how the app produced knowledge that they considered empowering (such as the disappearance of an old symptom). Yet, many also expressed ambivalences about how self-tracking would affect their everyday life. This suggests to us that hopes about distancing illness through the ease of use were not always realized. One interlocutor explained that by visualizing the symptoms, the app made her realize that she was practically always in pain: ‘I have sometimes used [the app], but the number of symptoms started to distress me, and I began to monitor them all the time. That’s why I don’t use it anymore’ (Written story, early 20s). This reflects the findings of de Boer et al. (2023), in the context of self-tracking in menopause, that some women may prefer not to track their symptoms as self-tracking makes symptoms feel ever-present.

In another example from our data, an interlocutor with endometriosis explained that when her symptoms are particularly bad, she opts for a commercial period-tracking app because it is less laborious:

It’s like if I’m too tired to add the symptoms [in the patient organization’s app]. Because unfortunately there are symptoms, there are days when I have to wear maternity clothes because my stomach swells so much. Sometimes I feel that I don’t want to think about [all the symptoms] I’ve had today. Then I might just add things in [the commercial app]. (Interview, mid-20s)

However, there is also affective work involved in using a commercial app. The same interlocutor explained that the commercial app ‘has been notifying me for the past half a year that hey, is everything okay, should you see someone because your period is 14 days long and that’s not normal?’ (Interview, mid-20s). While, for this interlocutor, tracking symptoms in the specialized gynecological app may have felt overwhelming, the commercial app is built around assumptions about normal cycles that can appear irrelevant for someone with endometriosis, highlighting their physiological difference from the idea of a healthy body. The example demonstrates how expectations about what technologies can achieve are relational in everyday use, as users consider the pros and cons of specific technologies in relation to their personal, situated priorities (Della Bianca, 2021; Ford et al., 2021; Pantzar & Ruckenstein, 2017). The differences in views of laboriousness and invasiveness across our data show that the patient organization’s app is mobilized in relation to other available options, most importantly the paper-based symptom diary and commercial period tracking apps. The temporal trajectories of chronic, potentially progressive illness—trajectories that differ based on endometriosis types and individual responses to medications—set the parameters within which situated choices between tracking methods are made.

While our data includes considerable variation in how daily tracking of endometriosis symptoms is viewed, there was one context in which the app was seen across patient advocacy, clinical care, and everyday experiences as particularly useful: seeking and establishing endometriosis diagnosis and treatment. As mentioned earlier, from the beginning of the design process, expectations of the patient organization were focused on the clinical use of the app. To this end, the organization collaborated with specialist clinicians to decide which symptoms should be included to facilitate earlier diagnosis. The possibility of downloading overview information of symptoms, indicating how many days per month users had symptoms such as pain, was a central design feature of the app. According to a patient organization representative, the idea was that you ‘can show it during your doctor’s visit’ and that way ‘have it on paper that you had symptoms then and then’. She noted that the app also includes ‘a possibility to follow whether you’ve been away from work or school, or considered being absent, due to the symptoms’ as well as mark down pain medications taken to alleviate the symptoms—information that could be used in diagnosis and decisions about treatment.

While assisting clinicians in diagnosis and treatment, the tracking of symptoms was also expected to help people with endometriosis to understand the nature and extent of their illness. This is particularly important in the case of healthcare encounters with clinicians who do not specialize in endometriosis. Wilkinson (2020) notes in the context of ovulation biosensing that ovulation tracking kits help make ovulation tangible, thereby making women feel more confident when meeting a clinician. Likewise, the patient organization envisioned that the self-knowledge engendered through the app would strengthen a patient’s position when seeking care for gynecological symptoms.

This role of the app in diagnosis and medical care was seen as important by many of our interlocutors with endometriosis. In such cases, the expectation of the users corresponded with the expectations of the patient organization. For example, one interlocutor told us that the app made her realize that she was ill 27 days out of 30:

[The app] was a really important part of my life when it was suspected that I might have endometriosis, and I was waiting for an appointment to a gynecology clinic. I was able to better read my body and how it worked. I also saw concretely the days with pain, which were in the worst case 27 days out of 30. Chronic pain was so normal that I had learned to live with it. (Written story, mid-20s)

For this interlocutor, the app moved pain from the realm of experience and the blurry world of not-quite-normal to concrete numbers that showed how all-encompassing and clearly abnormal the pain had become. This increased self-knowledge enabled by the app constituted a central step on the interlocutor’s path toward diagnosis and treatment.

Furthermore, some of our interlocutors with endometriosis mentioned that the app allowed them to make sense of the complex entanglements of symptoms and medication. For them, the usefulness of monthly overviews extended beyond diagnosis to the medical management of illness, broadening the horizon of expectations around the app. In a study on self-tracking and HIV care, Marent et al. (2018) note that some of their interlocutors found it useful that an app would remind them when to take medication. An HIV app was seen as having a role in preventing or managing serious illness. In our study, the patient organization’s app was experienced among some users as providing a sense of order to the interplay of medications and endometriosis. For example, one interlocutor, who had used the app for a little over a year, told us: ‘When you are tired or foggy from medication you really can’t always remember what medications you have taken, that’s why I mark in all the pain medications I take’ (Written story, early 30s). For another interlocutor, the app provided a potential means of tracking whether new medications work:

When I started a new pill in January, I was thinking how I have become blind to my own symptoms and pains. So, when the gynecologist said that the next appointment will be in April or May except if the symptoms get worse you can come earlier, my reaction was that I don’t know if they are getting worse. So, then I started using [the app] again and now I have used it quite actively since mid-January. (Interview, early 20s)

This suggests that there are situations in which the app is more than a tracking tool and may become a part of the apparatus of drafting and adjusting treatment paths for uniquely functioning bodies. In such situations, the goal of enabling personalized care through the app appears to be realized. The visions of the patient organization and the experiences of some of the users of the app are aligned.

While the patient organization’s app project drew on advice from specialized clinicians treating endometriosis, our interviews with people with endometriosis suggest that there is considerable variation in how healthcare professionals outside this group of specialist doctors view the app. Based on such descriptions of clinical encounters, it appears that many non-specialist clinicians had few if any expectations about the app. The situation reflects the dispersion of the Finnish healthcare system, as people with endometriosis may seek help with general practitioners in public healthcare clinics, nurses and doctors in student health services, gynecologists at private clinics and, through referral, gynecologists at specialized public endometriosis clinics. As the expertise on endometriosis varies considerably between these clinical settings, so do clinicians’ awareness and views of the app. Some of our interlocutors with endometriosis have positive experiences of showing the data to the doctors, as in this description of a follow-up visit: ‘When I saw the gynecologist this week, I used the app to show all the symptoms I’ve had … so perhaps it made it easier to evaluate whether the pills are working’ (Interview, early 20s). Yet, many note that they have met doctors who did not pay attention to the symptom overviews they had printed from the app: ‘I had printed several months of statistics, how bad the situation was, but the doctor wouldn’t even look at it’ (Interview, mid-20s). These differences between experiences point to the uneven integration of newly promoted technologies from outside the medical community into the institutionalized structures of clinical knowledge production. The situation resonates with recent findings by Haase et al. (2023) in Denmark, showing that many clinicians do not consider patient-provided digital information as clinically meaningful. Likewise, Ruckenstein (2015) has noted that the use of a digital app in a clinical context can create unease among clinicians when they are presented with data that does not fit into the parameters of standardized clinical data collected during medical appointments and examinations.

Not surprisingly, the app was relatively well-known among the healthcare professionals specialized in endometriosis who we interviewed. Our clinician interlocutors told us that they mention the patient organization and its peer support materials to patients at the time of their diagnosis. Healthcare professionals specialized in endometriosis stressed how the app is useful in that ‘it asks about the symptoms monthly, which is really good’ and ‘it’s good that the patient also sees other symptoms than just pain’. A specialized endometriosis nurse asserted: ‘All women should track their menstruation, when you get a period, whether it’s a symptom diary or calendar. And in endometriosis it’s good to mark down bleeding, pain and symptoms.’ Yet, we also heard clinicians question whether digital self-tracking is always needed. Some healthcare professionals mentioned that self-tracking might lead to patients focusing on their symptoms too much. For example, one sexual counselor specializing in gynecological conditions including endometriosis noted:

Sometimes I think it becomes never-ending, they monitor the body too much, every instance of pain, and then carefully report that. My work is to reduce some of that, not to self-track all the time because it makes self-tracking the focus.

This example suggests that if the patient’s treatment plan is based on drawing the attention away from constant pain, careful measurements of pain symptoms might be seen as a hindrance to care. Such differences in views among healthcare professionals make visible that expectations also reflect clinical specializations, such as gynecology or sexual counselling. In the case of gynecology, the self-tracking app can help to evaluate the symptoms prior to diagnosis as well as monitor how endometriosis responds to treatment, while in sexual counselling, the aim is to learn to cope with the pain.

## Structural challenges shaping expectations about use

Even when a new technology is viewed positively across patient advocacy, clinical care and everyday experiences of illness, expectations about how it could and should be used vary. For example, the usefulness of the app in endometriosis care was evaluated differently when mobilized as a technology specifically designed to capture endometriosis symptoms, as a means of making sense of embodied experiences of illness, or as a tool assisting in clinical assessment. In this final analysis section, we turn to one area where expectations were not easily aligned: the potential integration of the app into healthcare infrastructures. Such integration would mean moving beyond self-printed charts shown by the patient to their healthcare provider towards self-tracking data travelling through digital pathways between the patient, the healthcare system, and biomedical research institutions. This example sheds light on how expectations are entangled with and shaped by structural conditions.

When developing the first prototype of the app, the patient organization realized that the app had wider potential than initially anticipated. In the words of a patient organization representative: ‘I feel that we all realized only at that point its big potential. That this is not just for young people, but it has also huge possibilities for research, it gives huge help for everyone.’ When the organization applied for and received new funding in 2019, new goals were added to the project, including potential integration of the app into the digital health services in Finland, as well as enabling biomedical researchers to use the app in the study of endometriosis. Initiatives to integrate self-tracking tools into clinical and research infrastructures are not novel. For example, clinician-researchers have been interested in finding ways of integrating patient-produced menstrual symptoms data into biomedical research settings. Such data is envisioned to provide valuable information about menstrual variation and the connection between menstruation-related symptoms and various health conditions (Houghton & Elhadad, 2020). What is unique about our case, however, is that the app was designed by the patient organization rather than commercial technology companies. The patient organization also played a key role in pushing for the integration of the app. The idea for the integration of the app into clinical and research infrastructures evolved from the expectation that the app would provide important data to help diagnosis and treatment. As the potential of the app became increasingly clear during the design and testing of the prototype, the patient organization continued to be in active contact with specialist clinicians. These discussions concerned finding a concrete solution to how the app could become a part of the digital health services and research infrastructures.

While the practices of self-tracking are aligned with the widely shared policy aim of personalizing biomedical care, previous literature has documented that the integration of new technologies into the infrastructures and epistemic frameworks of biomedicine faces challenges (Vegter et al., 2021). Our case study adds to this literature by showing that integration is particularly difficult when the technology is developed by a patient advocacy group without formalized relations to the healthcare providers. One challenge the patient organization’s app encountered concerned the transfer of data between the app and healthcare institutions. As mentioned in the previous section, the users can currently print out the overview of their symptoms from the app and show it during the clinical visits. Integrating the app into the clinics’ digital health infrastructures would allow direct transfer of symptoms data from the app to healthcare records to, for example, streamline the diagnostic process. One patient organization representative elaborated on this vision:

The doctor could get some of the information even before the consultation. If you consider a situation in which a person goes to primary care with a suspicion and then they are usually referred to further evaluation. At that point, when asked for background information [for that second visit], they [healthcare providers] could have certain information—with the app, they could, for instance, ask you to use it until the next visit.

While pre-consultation data collection is already an established part of clinical work, as patients are asked to fill out online forms before their appointment, the patient organization envisioned that the app could ease the gathering of information about individual symptoms as well as support patients’ narration of the severity of their symptoms.

The biggest challenge in the integration of the app into the healthcare systems has been the need to protect patient information. According to a patient organization representative we interviewed, it is crucial that the user would still have the right to govern what data is shared with clinicians. However, digital healthcare systems have their own rules and logics of data ownership. Another patient organization representative noted:

We have developed this ourselves and I don’t see that [the patient organization] can let go of the ownership. We cannot just give this away. We give this for everyone to use free of charge and if we sold this to a hospital group or gave the governing or ownership rights to someone else, we would jeopardize that.

Expectations about integrating the app into digital healthcare infrastructures thus clashed with a key premise of the entire app development project: The app differs from commercial apps in that it prioritizes the rights and wishes of the users.

Issues of security and privacy were seen as the biggest challenge from the viewpoint of hospitals, as well. However, the healthcare providers view security and privacy from a different angle. According to one of the clinicians we interviewed, the challenge for them is that the app did not employ the standard security measures used in the healthcare system.

Maybe the biggest problem why we couldn’t integrate it into the digital services are these kinds of boundaries between the hospital information system and outside systems. The connection between them needs to be safe, which means that you would have to have a strong form of identification to log into [the app]. And you don’t have that—logging in with your bank identifiers [the established form of secure identification for healthcare and public services in Finland]. And it would have required a lot of work and money to integrate it into the hospital patient record systems.

This example suggests that any new technology developed outside the institutional structures of healthcare would have to be amended to fit the established institutional practices of data collection and transfer. Only then could hopes and visions about integration be realized. The quotation also shows that both further development and funding would be needed for the integration to take place, a point that was also emphasized in the patient organization representative interviews. This can be challenging because of the lack of funding for endometriosis, and many diseases associated with women more generally (Ellis et al., 2022). Together these interviews highlight that while the patient organization and the clinicians shared the view that integrating the app would be challenging, both sides also had their own requirements for the app: the free use and governance of data for the patient organization and a standardized form of log-in for the hospitals.

Another challenge for the app’s clinical use was its adaptability for medical research. The app can be used for research data collection, and it was used as a part of an epidemiological follow-up study to identify how many people showed signs of gynecological conditions. All participants agreed to this separately. However, our clinical interlocutors also mentioned challenges in using the app for research. The biggest issue they identified was the lack of official integration into existing digital healthcare infrastructures as described above. Furthermore, even if the app were integrated into the digital healthcare infrastructures in the ways envisioned by the patient organization, the collected data might not meet the standards required for medical research. One clinician we interviewed mentioned that in endometriosis research ‘the patient should mark down daily menstrual flow, pain level and pain medications, but [the app] asks much more than that’. Furthermore, the clinician noted a difference in the measuring scales for pain:

If a study uses, for example, a pain symptom, we’d need to estimate the strength of the pain from one to 10, which is the right way to measure in research. And in [the app] you have a five-step scale, so we couldn’t use [the app] directly in research.

Self-recorded data does not necessarily fit into established biomedical practices (Ruckenstein, 2015). While the two ways of scaling pain both fulfil the function of estimating the severity of pain, the established 1-to-10 pain scale used in clinical practice has shaped the requirements for clinical research. Patient advocates’ hopes of expanding the app’s use in endometriosis research were curtailed by the discrepancy between a measuring system designed to be accessible and easy for the app user and one that aligns endometriosis symptoms with a broader biomedical framework of diseases and symptoms. Since these interviews, the app has implemented the scale of 1 to 10 for pain symptoms, making the app more compatible with clinical practices.

Despite the popular idea that technology will connect sites and make data flow, the challenges documented here suggest that it is difficult to make data, gathered through an app outside the healthcare infrastructures, flow across sites. In the case of this app, these interruptions in data flow are a matter of differences between systems. The systems in question involve different expectations about how to assign data ownership and control, as well as how to secure the identity of the user. While these are structural issues, they also involve values and priorities. Expectations also come with different intensities: While clinicians may view the app as a practical and convenient tool, patient advocates invest in it a broader set of hopes and visions. Furthermore, the case demonstrates how the flow of data presumably achieved by technological innovation can be hampered by the incompatibility of different ways of measuring. What constitutes a meaningful, carefully thought out means of tracking pain in daily use may not be easily incorporated into standardized research infrastructures relying on institutionalized conventions of measuring pain. Crucially, the requirement for standardization does not indicate that one system of measuring is better than others. Rather, it reflects practical concerns arising from epistemic conventions within biomedicine. The standardized biomedical scale may not correspond with embodied experiences of illness any better than other scales, and it may contradict the principle of ease of use that guided the design of the app. While our case study shows that the app is open to redesign, the differing pain scales exemplify the challenges that may occur when implementing a patient-advocacy led technology to clinics. The premises guiding the app design can be difficult to merge with the highly standardized and often conventional expectations of technology use in clinical and research settings.

## Conclusion

The article set out to investigate the possibilities and limits of patient advocacy-led technology development in a complex and under-researched chronic illness. To this end, we have explored the evolving dynamics of expectations around a gynecological self-tracking app developed by a Finnish patient organization to advance endometriosis care and research. We have situated the app in relation to commercial period tracking apps as well as self-tracking in chronic illness. Our analysis focused on three questions around the use of the app in endometriosis. First, we asked to what extent expectations about the app’s ability to capture endometriosis symptoms were realized. The app expanded ideas of what can be traced in relation to bleeding and hormonal changes, establishing links across gastrointestinal symptoms, shifts in the type of pain, use of medications, and ability to work or attend school. At the same time, the need to consider ease of use in design left limited space for symptoms that are uncommon yet—due to the complexity of endometriosis as an illness—may be central to a person’s illness experience. Furthermore, while the app produced a sense of chronic temporality that departed from the cyclicality of commercial period tracking apps, the pattern-oriented logic of digital self-tracking did not easily capture experiences of chaotically undulating symptoms.

Second, we examined what different uses were imagined for and expected of the app, and how these visions changed at different sites. Our analysis shows that the app was envisioned by the patient organization as making symptom tracking less laborious than paper-based diaries and more accurate than commercial period tracking apps. The expectation that the app would reduce the need to dwell on symptoms was received with some ambivalence: many of our interlocutors welcomed the idea while others expressed doubts. All in all, in the interviews with and stories by people with endometriosis, situated uses of the app emerged. These ranged from understanding the realness of the illness to tracking the effects of medication and observing gradual changes in the condition. While the experiences of the users varied greatly, our interlocutors across patient advocacy, clinical settings and everyday experiences shared the expectation that the app could assist in gaining diagnoses and in tracking the effects of treatment. However, the realization of this expectation in different healthcare settings appeared to be inconsistent.

Third, we asked how structural aspects of technologies and technological systems shaped expectations about the future of the app. We identified the app’s potential integration into the digital infrastructures of healthcare and biomedical research as an area where the ideal of data flowing smoothly across sites could not be realized as hoped by the patient organization. One promise of quantification is that knowledge can be transported across sites. However, in our data, differing views of how systems should be interlinked generated tensions in expectations that seemed previously aligned. In particular, concerns about data ownership and security of identification shaped how expectations about the app have evolved, respectively, in patient advocacy and in specialized clinical settings. The case demonstrates the difficulty of bringing a patient-advocacy led technology into established institutional infrastructures, even when the involved parties share a vision about the technology’s relevance. The patient advocates and healthcare professionals we interviewed highlighted that addressing these infrastructural challenges requires considerable time and funding. This can be challenging as endometriosis and other gynecological conditions remain underfunded, as mentioned by both our clinical and patient advocacy interlocutors. These challenges point to the kinds of difficulties that other patient advocacy-based health technologies may encounter when addressing other illnesses, especially ones that are underfunded and under-researched.

This case shows that a range of possible scenarios open up and subsequently close as a patient advocacy-led technology is designed, launched, and used. It shows that actors involved in patient advocacy, clinical settings and everyday practices of living with a chronic gynecological illness attach different expectations to these moments of opening and closing. For example, what is a flagship project for the patient organization is inevitably one among other measuring and tracking technologies for those working in healthcare settings. Our data shows that while clinicians specializing in endometriosis saw the benefits of the app and recommended it to their patients, they could also be hesitant when considering its further use in treatment and research, mentioning different rationales of measuring and accessibility policies as main reasons. This shows that while issues such as structural or financial constrains may shift expectations across the contexts involved in the design and use of the technology, expectations may not shift in the same direction.

Despite these differences in expectations, the patient organization’s app has been in many ways successful. Crucially, the app was enabled by the organization’s long-standing work to establish itself as a reliable actor, trusted by both people with endometriosis and clinicians specialized in endometriosis. Such collaboration also helped to minimize the gap between the symptom information tracked in the app and the symptom information relevant for diagnosis and treatment. Our analysis shows that the material conditions around the app’s development—targeted project funding, established collaborations with clinicians, different digital and patient data platforms—ultimately shaped how the app could be used to advance endometriosis care and research. Furthermore, the app design project relied on a vision of digitization as a key area in managing and treating chronic illness shared with many healthcare professionals as well as people using the app. Through these points of connection, the patient organization created a shared landscape of expectations that, despite differing emphases and emerging challenges, has carried the app project. While the app has not, by the time of writing this article, been integrated into digital infrastructures in clinical settings, it is widely recognized in healthcare settings specializing in endometriosis. The app is understood across our research contexts to provide an efficient, illness-specific mode of following the development of symptoms and the effects of medication beyond the normative framework of the average menstrual cycle.
